# MidFusionEfficientV2: Improving Ophthalmic Diagnosis with Mid-Level RGB–LBP Fusion and SE Attention

**DOI:** 10.3390/jcm15062352

**Published:** 2026-03-19

**Authors:** Julide Kurt Keles, Soner Kiziloluk, Eser Sert, Furkan Talo, Muhammed Yildirim

**Affiliations:** 1 Department of Eye Clinic, Elazig Fethi Sekin City Hospital, Elazig 23300, Türkiye; julide_kurt@hotmail.com; 2 Department of Computer Engineering, Malatya Turgut Ozal University, Malatya 44200, Türkiye; soner.kiziloluk@ozal.edu.tr (S.K.); eser.sert@ozal.edu.tr (E.S.); 3 Department of Computer Engineering, Firat University, Elazig 23119, Türkiye; ftalo@firat.edu.tr; 4 Department of Artificial Intelligence and Data Engineering, Firat University, Elazig 23119, Türkiye

**Keywords:** eye diseases, deep learning, EfficientNetV2-S, Local Binary Pattern (LBP), Squeeze-and-Excitation

## Abstract

**Background/Objectives**: Early diagnosis of eye diseases is critically important for enhancing individuals’ quality of life and reducing the risk of vision loss. In this study, a deep learning-based hybrid model called MidFusionEfficientV2 has been proposed to classify eye diseases, including uveitis, conjunctivitis, cataract, eyelid drooping, and normal conditions. **Methods**: The model presents a dual-branch architecture that combines an RGB image branch with an EfficientNetV2-S architecture and a specialized texture branch based on Local Binary Pattern (LBP) transformation at an intermediate level. Thanks to the Squeeze-and-Excitation (SE) blocks integrated into the LBP branch, channel-based attention mechanisms have been activated, enhancing the prominence of textural features. The features obtained from the RGB and LBP branches were combined at an intermediate level and transferred to the classification stage. **Results**: Experimental studies on the five-class eye disease dataset from the Mendeley Data platform have shown that the proposed model outperformed six strong models (ResNetV2, ConvNeXt, DenseNet-121, EfficientNet-B1, MobileNetV3 Large, and EfficientNetV2-S) with an accuracy of 98%. Especially in the difficult-to-diagnose uveitis class, recall and F1 scores of 97% and 94%, respectively, were achieved. **Conclusions**: The results show that a moderate combination of color and texture features significantly improves classification performance, and that MidFusionEfficientV2 offers a reliable and effective solution for the automatic diagnosis of eye diseases.

## 1. Introduction

Eye diseases such as uveitis, conjunctivitis, cataracts, and eyelid disorders are significant health problems affecting millions of people worldwide. These diseases can significantly enhance the patient’s quality of life if diagnosed early and treated appropriately. Nevertheless, conventional methods of clinical examination, as well as image analysis, are too slow, expensive, and heavily reliant on the unique skill of other doctors. There is a greater need for automated healthcare decision-support -systems, particularly in areas with limited medical services. Artificial Intelligence (AI) and Deep Learning (DL) responsive systems are very effective in dealing with this lack [1,2].

In recent years, DL-based image analysis systems have demonstrated significant success in the medical field. Literature research demonstrates that Convolutional Neural Networks (CNNs) achieve high success rates in automatic feature extraction and classification from clinical images [2,3]. In a study by Wanto et al. [4], deep learning models such as VGG19, InceptionV4, Resnet50, MobileNetV2, MobileNetV1, MobileNetV3 Large, and MobileNetV3 Small were used to analyze retinal fundus images to detect various eye conditions such as diabetic retinopathy, drusen, and central retinal vein occlusion.

Deep learning methods have successfully classified many eye diseases using fundus and OCT images. Models that achieve over 99% accuracy in VGG19-based OCT classification have achieved 94–99% accuracy in automating retinal disease detection and classifying conditions such as macular edema, age-related macular degeneration, and diabetic retinopathy [2].

In the study conducted by Dash et al. [5], an approach based on a hybrid deep learning method for the detection of eye diseases using fundus images has been presented. In the relevant study, deep features extracted using the VGG16 architecture were obtained through images enriched with Grad-CAM and Multiscale Retinex (MSR). Then, these features were combined, and the k-nearest neighbor (k-NN) algorithm was used to classify eye diseases. While an accuracy of 89.2% was achieved with only Grad-CAM and 83.1% with only MSR, a combination of the two resulted in an accuracy of 96.5%. However, most of the approaches in the literature rely solely on color and positional information, whereas the complex tissue information in medical images plays a critical role in the diagnostic process.

Local Binary Pattern (LBP) is an important approach used to digitize complex texture information in images effectively and at a low cost. LBP has been used in various studies to enhance tissue-based classification performance in medical images [6]. However, in the literature, the innovative integration of LBP with next-generation deep learning architectures is still limited; yet it is a well-known fact that hybrid models enriched with texture information show performance improvement [7].

EfficientNet [8] and its successor, EfficientNetV2 [9], are successful CNN architectures that achieve high accuracy with limited parameters thanks to compound scaling and automatic architecture search. EfficientNetV2 S, in particular, has attracted attention with its fast training time and high generalization ability in the field of medical image classification [10]. Furthermore, EfficientNetV2-based models have also played a role in the diagnosis of diseases such as diabetic retinopathy and macular degeneration [11]. Sebastian et al. [12] investigated DL-based methods for the early diagnosis of Diabetic Retinopathy, a disease that occurs due to diabetes and can lead to vision loss. This study focused specifically on automatic diagnostic systems developed using fundus images. This study summarizes existing deep learning approaches, frequently used datasets, and the performance of these methods by comparing them.

The Squeeze and Excitation (SE) block, which provides a channel-level attention mechanism, has been frequently used recently. SE, which has a modular structure, is computationally efficient. If SE blocks are integrated into the initial layers of the network, they can enable more successful results in extracting general features such as textures and edges. In deeper layers, SE blocks can develop class-specific high-level semantic representations [13]. These blocks, due to the adaptability they provide throughout the network hierarchy, can enhance feature differentiation and overall model accuracy, particularly in the classification processes of images containing small details. In summary, SE blocks are used in various studies to improve the performance of CNN models [14,15].

In addition to CNN-based studies, Vision Transformer (VIT)-based models have also been frequently used in the diagnosis of eye diseases in recent years and have yielded successful results [16]. However, one of the most significant disadvantages of VIT-based classification systems is their need for large data sets and time-consuming training processes.

In our MidFusionEfficientV2 model, developed for the detection of eye diseases, we created a hybrid model by combining the EfficientNetV2-S backbone, an LBP-based subnet, and the SE Block module. The developed MidFusionEfficientV2 model achieves more successful performance metrics by using color and texture information together. The developed model aims to address a significant deficiency compared to classical RGB-based CNN approaches. The innovative aspects of this study and its contributions to the literature are presented below.

- With the Mid-Fusion Dual Branch model global features derived from RGB images and local texture features obtained from LBP transformation are processed simultaneously, and an innovative structure is created thanks to the design that combines these two separate branches at the Mid-Fusion level.
- To process LBP-transformed images, a separate CNN-based LBP branch has been developed, highlighting tissue features in medical images.
- To enrich texture features, a channel-level attention mechanism is provided using SE blocks integrated into the LBP branch, aiming to make the texture features extracted from LBP more meaningful and distinctive.
- With optimized training with differential learning rates, the pre-trained backbone was fine-tuned by assigning different learning rates to different model branches. In contrast, branches trained from scratch were trained more aggressively and efficiently.
- In terms of the impact of using LBP-based texture information on classification performance compared to traditional RGB-based models, the MidFusionEfficientV2 approach, which combines LBP-based texture features at a moderate level, achieved superior classification accuracy compared to EfficientNetV2-S and other CNN models.

## 2. Literature Review

With the advancement of technology, the number of studies in the literature that use architectures such as LBP and CNN in the field of image processing is increasing. Zaeri and Dib developed an approach that combines angular and radial LBP for diagnosing pneumonia in X-ray images. The model proposed by the researchers achieved an accuracy rate of 82.7% [17]. Kumar et al. proposed a model for diagnosing oral cancer that combines feature maps from LBP and ResNet50. The researchers tested the proposed model on a dataset of 3000 oral cancer images and achieved 98% accuracy [18]. Hossain et al., in their study on the classification of kidney abnormalities, used the Adaptive LBP technique to extract features from a CT dataset. These features were then classified into five different classifiers. The researchers then used a soft voting method and achieved an accuracy rate of over 99% [19]. Gul proposed a model called QS-LBP for breast cancer detection using histopathological images, employing an LBP-based and 20-layer CNN architecture. The proposed QS-LBP model achieved an accuracy rate of 94.58%, while the proposed 20-layer CNN architecture achieved an accuracy rate of 98.27% [20].

Gul and Kaya used different LBP techniques for brain tumor classification [21]. In the proposed method, they first performed feature extraction using these three different LBPs and then classified these features using 11 different machine learning classifiers. In the experimental study, the instance-based learner algorithm yielded the best result with 99.12%. Garg et al. [22] proposed an approach using wavelet transforms and the Shifted Elliptical LBP algorithm for the diagnosis of Alzheimer’s disease from magnetic resonance images (MRIs). The study achieved an accuracy rate of 96%. Alabdulqader et al. developed an LBP-based method integrated with the EfficientNetB4 model for classifying colon and lung cancer images. This model, developed using EfficientNetB4 and LBP, achieved an accuracy rate of 98.8% [23]. Singh and Singh proposed an LBP-based transfer learning method for the diagnosis of lung and colon cancer. In this study, LBP was used for feature extraction and the ResNet50 model for classification. The proposed model detected lung and colon cancer with a 99% accuracy rate [24].

Many studies integrate attention mechanisms into deep neural networks. Attention mechanisms enable models to capture important information by weighting features at the channel level. Gencer and Gencer developed a hybrid model by combining EfficientNetB0 and Xception architectures with SE blocks to detect eye diseases such as choroidal neovascularization (CNV), drusen, and diabetic macular edema (DME) from Optical Coherence Tomography (OCT) images. The researchers tested the proposed model on Duke’s OCT and UCSD datasets, achieving an accuracy of 99.58% [14]. In a separate study using a similar dataset, Mori and Modi developed a model known as SeResNet that integrates SE blocks with traditional residual networks. The researchers tested their proposed model on an OCT dataset with CNV, drusen, DME, and normal classes and achieved a 99% accuracy rate [25].

Assaduzzaman et al. [26] proposed a model called XSE-TomatoNet for the classification of tomato leaf diseases. The proposed model was built on the EfficientNetB0 architecture and supported by SE blocks. In the experimental study, 11 different types of tomato diseases were classified with 99.11% accuracy. Wu et al. [27] proposed a model called ResNet9-SE, which combines the ResNet architecture with the SE block for disease detection in strawberry plants. The proposed model classified strawberry diseases with 99.7% accuracy. Siam et al. [28] proposed a model called SE-VGG16 MaizeNet for the detection of corn leaf diseases. In the proposed model, they integrated the attention mechanism with SE blocks onto VGG16. In experimental studies, the SE-VGG16 model achieved 93.44% accuracy.

AbdelAziz et al. [29] proposed a model called SECNN-RF that diagnoses Alzheimer’s disease using MRIs. In their study, they combined a CNN model equipped with SE blocks with a Random Forest classifier. In experimental studies, the proposed model achieved 99.89% accuracy. Yuan and colleagues [30] proposed a model using SE-dilated residual blocks for Alzheimer’s diagnosis. The proposed model was able to predict the probability of conversion to Alzheimer’s within 6 to 36 months with 88% accuracy. Maheswari and Gopalakrishnan [31] developed the SE-Capsule Network architecture and performed disease classification on datasets consisting of X-ray, CT, and MRIs. The study also performed feature selection using the Woodpecker Mating Optimization algorithm. Experimental studies showed that the proposed model achieved 99% accuracy.

Vadduri et al. used both classification and segmentation techniques in their study. Their work employed histogram equalization steps and achieved class-based accuracy values in the 96–98% range [32]. Nguyen and Lin [33] proposed a model for the automatic diagnosis of cataracts by training it on full retinal images and quadrations with a hybrid CNN architecture. The model improved its performance by detecting small anomalies in the image through segment-based analysis and achieved an accuracy of 97.12%.

Syahira et al. used CNN architectures in their study to classify different eye diseases [34]. The model was evaluated on a dataset with six categories (cataract, uveitis, bulging, strabismus, glaucoma, and normal) and achieved 94% accuracy, particularly with the Adam optimization algorithm. Khalid et al. [35] proposed a method called CAD-EYE for multi-eye disease classification. In the study, they combined the features of MobileNet and EfficientNet architectures and aimed to improve interpretability with fluorescence imaging. They achieved 98% accuracy in the experimental study.

## 3. Materials and Methods

In this study, a deep learning-based method has been developed for the automatic classification of eye diseases (uveitis, conjunctivitis, cataract, eyelid drooping, and normal). The proposed method is a mid-level fusion model named MidFusionEfficientV2. This model combines an RGB branch based on EfficientNetV2-S, a convolutional branch that extracts Local Binary Pattern (LBP) features, and a compression-excitation (SE) block. The model aims to achieve high accuracy and generalization performance by combining both color and texture information. Below, the dataset, the LBP method, EfficientNetV2-S, other technical details (SE block, data augmentation, optimization techniques), and all stages of the proposed method are described in detail.

### 3.1. Dataset

In this study, the “Image Dataset on Eye Diseases Classification (Uveitis, Conjunctivitis, Cataract, Eyelid) with Symptoms and SMOTE Validation” dataset obtained from the Mendeley Data platform was utilized [36]. The dataset used in this study consists of JPEG images classified into categories such as uveitis, conjunctivitis, cataract, eyelid drooping, and normal. Each category is determined based on the presence of visual features and symptoms specific to the respective disease. The dataset was obtained from online sources and subsequently verified by the Bangladesh Eye Hospital. The dataset used in this study contains a total of 2298 images.

**Figure 1:**
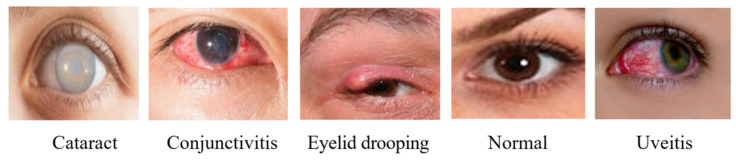
Example images for each class from the dataset.

- Normal—A normal eye is associated with the absence of abnormalities, redness, inflammation, or other pathological signs. Images classified as normal show the unobstructed, natural appearance of healthy eyes.
- Uveitis—This is a class of diseases characterized by various symptoms such as redness, pain, blurred vision, light sensitivity, and floaters in the eye. Irregularities and signs of inflammation are commonly seen in this category of diseases.
- Conjunctivitis—This class of diseases includes redness, itching, tearing, and discharge. The images in this category show marked redness and surface irritation of the conjunctiva.
- Cataract—In this class of diseases, symptoms such as cataracts, blurred vision, difficulty seeing at night, and light sensitivity are observed. The images in this category show the lens’s transparency and the presence of cloudy areas.
- Eyelid drooping—Sagging, inflammation, and irritation of the eyelids. The images depict tissue changes on the skin surface and eyelid deformities. Figure 1 shows sample images representing the classes in this dataset.

### 3.2. Local Binary Pattern (LBP)

Local Binary Pattern (LBP) is an effective method used to extract texture features from images [37]. LBP works by comparing a pixel with its neighboring pixels and creates a binary pattern as a result of these comparisons. Mathematically, the LBP code is calculated as follows in Equation (1).

(1) $$LBP\left(x_{c},y_{c}\right)=\sum_{p=0}^{P-1}s(g_{p}-g_{c})\cdot 2^{p},s\left(x\right)=\left\{\begin{array}{c} 1,\;x\geq 0\\ 0,\;x<0 \end{array}\right.\;\;$$

Here: (*x_c_, y_c_*), coordinates of the central pixel; *g_c_*, grayscale value of the central pixel; *g_p_*, grayscale value of the neighboring pixel; *P*, number of neighboring pixels (usually 8); *s*, sign function (provides binary coding of pixel differences).

In this study, the uniform LBP method was used (P = 8, R = 1, method = ‘uniform’). Uniform LBP generates 59 different patterns by considering patterns with a maximum of two bit transitions and is more robust to noise. Uniform LBP represents texture features in a more consistent and noise-free manner. The LBP method is particularly effective in extracting texture-based features (surface irregularities, skin patterns) such as uveitis and ptosis. For example, surface irregularities caused by uveitis or the opaque texture of cataracts can be captured with the LBP method. LBP strengthens the classification performance of the model by providing a feature set complementary to the color information obtained from RGB images.

### 3.3. EfficientNet and EfficientNetV2-S

EfficientNet is a family of CNN that offers high accuracy and low computational cost in deep learning-based image classification tasks [38]. EfficientNet uses a compound scaling method that scales the network depth, width, and input resolution in a balanced manner. This approach optimizes the following key dimensions:

Depth—The number of layers in the network increases the capacity to learn complex features; however, this also raises the computational cost.

Width—At this stage, the number of channels in each layer facilitates the simultaneous processing of additional features. However, this increases memory usage.

Resolution—The pixel dimensions of the input image allow for the capture of more detailed features. However, this feature also increases the computational load on the model, which is a disadvantage.

EfficientNet’s compound scaling formula is as follows in Equation (2):

(2) $$d=\alpha^{\phi },\;w=\beta^{\phi },\;r=\gamma^{\phi }$$

In the equation, *d* is the depth scaling coefficient, *w* is the width scaling coefficient, *r* is the resolution scaling coefficient, *α*, *β*, and *γ* are constant coefficients, and *ϕ* is the composite scaling factor controlling the model size.

From EfficientNet-B0 to EfficientNet-B7, different models are scaled by increasing the *ϕ* value. The Mobile Inverted Bottleneck Convolution (MBConv) blocks in the basic structure consist of the following stages:

Expansion Layer: The expansion layer increases the number of channels, thereby expanding the model’s representational power.

Deeply Separable Convolution: Uses depthwise convolution and pointwise convolution to reduce computational cost.

Compression Layer: Reduces the number of channels and applies channel-based attention with the SE block.

**Figure 2:**
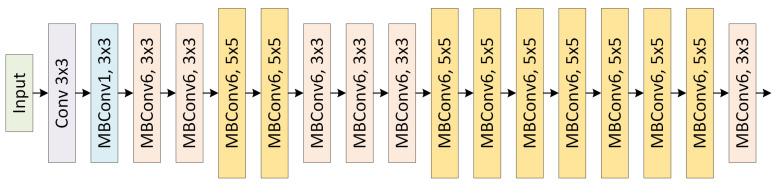
Architecture of EfficientNet.

The basic architecture of the EfficientNet model is shown in Figure 2 [38,39].

EfficientNetV2, an improved version of EfficientNet, was introduced in 2021 [9]. The EfficientNetV2-S model used in this study offers the following innovations:

Fused-MBConv Blocks: Expansion and depthwise convolution are combined into a single standard convolution. This particularly increases the training speed on GPUs.

Progressive Learning: During training, the image size and regularization parameters are dynamically adjusted. Training starts with small images and gradually transitions to higher resolutions.

Smaller Kernel Sizes: The computational cost is reduced by using 1 × 1 kernel sizes instead of 3 × 3.

Improved SE Blocks: Channel-based attention mechanisms are made more efficient.

**Figure 3:**
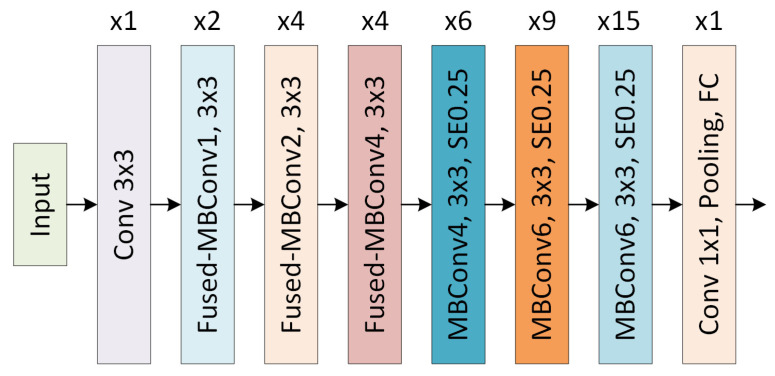
Architecture of EfficientNetV2.

The basic architecture of the EfficientNetV2 model is shown in Figure 3 [9,40].

In this study, EfficientNetV2-S is loaded with weights pre-trained on ImageNet from the timm library. Transfer learning enables the model to quickly adapt to eye disease images. In experimental studies, EfficientNetV2-S successfully detected opacity in cataracts, redness in conjunctivitis, and surface abnormalities in uveitis. However, it was observed that it was insufficient in extracting tissue-specific structural features, and therefore, fusion with LBP-based features improved classification performance.

### 3.4. Squeeze and Excitation (SE) Block

SE Block offers a channel-wise attention mechanism in deep learning architectures, thereby enabling the network to learn meaningful channels in feature maps more effectively [41]. SE enables the network to focus on more prominent features by determining the importance of channels. The SE block significantly increases the representational power of models, especially in classification and object recognition applications. The SE block consists of two stages.

1-Squeeze Stage: The spatial dimensions (height H and width W) of each channel are reduced to a 1 × 1 dimension using a global average pooling operation. In this step, the channels are transformed into a value that represents the average of all their spatial positions. This process summarizes the overall activation information that each channel carries throughout the image. This process can be expressed as in Equation (3).

(3) $$z_{c}=\frac{1}{H\times W}\sum_{i=1}^{H}\;\sum_{j=1}^{W}x_{c}(i,j)$$

In Equation (3), *z_c_* represents the compressed value of the *c-th* channel, and *x_c_*(*i*,*j*) represents the activation value of this channel at the *i*,*j* position.

2-Excitation Stage: In this layer, compressed values are passed through two fully coupled layers. The first layer performs dimensionality reduction by dividing the number of channels by 16, and ReLU activation is applied in this step. The second layer returns the number of channels to their original size, and the weights are normalized with sigmoid activation. The relevant operations are presented in Equation (4).

(4) $$s=\sigma (W_{2}\cdot \delta \left(W_{1}\cdot z\right))$$

In Equation (4), *σ* represents the sigmoid function, *δ* is the ReLU function, and *W*_1_, *W*_2_ denote the weight matrices of the two fully connected layers.

## 4. Proposed Method

The proposed MidFusionEfficientV2 model is a mid-level fusion approach that combines RGB and LBP features to classify eye diseases (uveitis, conjunctivitis, cataract, eyelid drooping, and normal). The model integrates the EfficientNetV2-S-based RGB branch, the convolutional LBP branch, and the SE block. A detailed description of the method is presented below.

### 4.1. Preparation of the Dataset and RGB Image Preprocessing

RGB images were adapted to the model in two sub-steps: data augmentation and normalization. Data augmentation is done to improve a model’s generalization capabilities and mitigate overfitting. With medical images, conditions such as lighting, angle, and the device used can cause variances that impact model efficacy. As a result, the following augmentation approaches were applied in this study:

Resizing: Images were augmented to a height and width of 224 and 224 pixels, which is the input dimension of the EfficientNetV2-S model.

Random horizontal flip: Images were flipped horizontally on random basis. As a symmetric image, eyes would not impact model performance.

Random Rotation: Images were randomly rotated to a maximum of 10 degrees to simulate minimal angular shifts.

Color Jitter: Changes in lighting conditions were simulated by adjusting the brightness, contrast, and saturation by 20%.

Overcoming model overfitting, data augmentation helps fortify the model’s performance on constrained datasets [42]. In the context of medical images, data augmentation allows the model to learn to simulate different conditions. In this way, the images are subjected to random transformations during the course of training.

RGB images were normalized according to the mean ([0.485, 0.456, 0.406]) and standard deviation ([0.229, 0.224, 0.225]) values of the ImageNet dataset. Normalization standardizes the distribution of input data, increasing the convergence speed of the gradient descent algorithm and strengthening the model’s stability. Normalization stabilizes the learning process in deep learning models, and ImageNet normalization ensures compatibility with pre-trained models for transfer learning [43]. With normalization, inputs compatible with the pre-trained weights of the EfficientNetV2-S model are obtained.

### 4.2. LBP Image Generation and Preprocessing

LBP is a method for extracting texture features and is effective in capturing the superficial texture differences of eye diseases (e.g., cloudiness of cataracts or inflamed tissue of uveitis). This step consists of three substeps.

RGB images have been converted to grayscale to focus on texture analysis. This process removes the color information, preserving only the intensity information, thereby providing a suitable basis for LBP computation.

### 4.3. Feature Extraction with EfficientNetV2-S

The RGB branch extracts high-level visual features (edge, shape, color patterns) using the EfficientNetV2-S model. EfficientNetV2-S is a convolutional neural network that has been pre-trained on ImageNet and is utilized for transfer learning [9]. The Mobile Inverted Bottleneck Convolution (MBConv) and Fused-MBConv blocks, which are the building blocks of the EfficientNetV2-S model, provide high efficiency and accuracy in extracting visual features [44]. These blocks enable EfficientNetV2-S to be both computationally optimized and capable of learning complex visual patterns (e.g., lesions in the eye or blurred areas in cataracts).

MBConv blocks incorporate an SE mechanism that emphasizes important features and suppresses unimportant ones by applying a channel-based attention mechanism. For example, prominent features such as redness in uveitis images are given more weight through the SE mechanism. Fused-MBConv blocks are a variant of MBConv where 3 × 3 convolution replaces depth convolution. The use of these blocks makes the model computationally efficient and allows the model to learn complex images more easily, such as the appearance of cataracts or the shape of a drooping eyelid.

Adaptive average pooling downsamples a feature map to a fixed size and enables the model to handle inputs of varying lengths [45]. The spatial information in the feature maps is compressed by this process to generate a fixed-size feature vector required for the subsequent fusion and classification phases. In the EfficientNetV2-S branch, the feature maps are pooled using adaptive average pooling to a 1 × 1 size, yielding a 1792-dimensional vector. This averaging operation transforms the two spatial dimensions (height and width) of each feature map into a scalar by averaging across the spatial dimensions while keeping the spatial resolution. In the LBP branch, feature maps are downsampled to 4 × 4, then flattened to a 1024-dimensional vector. These dimensions correspond to the two branches’ feature representations: the RGB branch models higher-level, general visual features, and the LBP branch models more local, texture-related features.

### 4.4. LBP Branch Feature Extraction

The LBP branch uses a specialized convolutional network to extract texture features and consists of four sub-steps. Capturing superficial texture differences (e.g., the blurred texture of cataracts, the inflamed areas of uveitis, or the redness patterns of conjunctivitis) is critical in the classification of eye diseases. For this purpose, a specialized CNN branch was designed for extracting LBP based features. The LBP branch consists of three convolutional layers, a SE block, adaptive mean pooling and smoothing steps to extract texture features and complement the visual features of the RGB branch. This branch effectively represents texture patterns in medical images, improving the diagnostic accuracy of the model. LBP extracts texture features by intensity comparison of a pixel with its surrounding neighboring pixels. Mathematically, LBP is expressed by Equation (1) given in Section 3.2.

Uniform LBP decreases computing complexity by focusing solely on patterns with a maximum of two bit transitions, hence offering a resilient representation against noise [46]. The output of Uniform LBP is derived from grayscale-converted images and normalized to the range of 0–255 for input into the convolutional network. The first convolutional layer of the LBP branch generates 16 output channels from the single-channel LBP image using a 3 × 3 kernel with a padding of 1 pixel. The second layer captures more complex texture features by switching from 16 to 32 channels. The third layer increases from 32 to 64 channels and produces more abstract, high-level texture representations. Each layer is stabilized by batch normalization, highlighting nonlinear features with the ReLU activation function, and halving the spatial dimensions with max pooling (2 × 2). Batch normalization improves model stability by reducing the internal covariance shift during the learning process [47], while ReLU enables the model to learn complex patterns by preserving positive values and zeroing out negative values [48]. Max pooling reduces the computational overhead by reducing the size of feature maps and supports the extraction of more abstract features. After the third convolutional layer, an SE block is applied. The SE block provides a channel-by-channel attention mechanism and highlights important texture features.

The SE block compresses the feature maps with global mean pooling, then computes channel weights with two fully connected layers (64 → 4 → 64) and sigmoid activation. These weights are multiplied by the feature maps to highlight important channels (e.g., uveitis-specific patterns of inflammatory tissue) [41]. Consequently, the feature maps are reduced to 4 × 4 dimensions by adaptive mean pooling and smoothed to produce a 1024-dimensional feature vector. Adaptive mean pooling involves compressing the spatial dimensions to a fixed size, thereby enabling the model to adapt to varying input dimensions. This configuration effectively extracts texture features from LBP images and complements the visual features of the RGB branch, thereby strengthening the model’s generalization ability [49].

In this study, the Uniform Local Binary Pattern (LBP) method was used to extract texture features. The LBP parameters were set to P = 8 adjacent pixels and R = 1 radius. This configuration is one of the most commonly used in texture-based image analysis studies in the literature. Uniform LBP offers advantages such as greater noise resistance and lower-dimensional feature representation by considering patterns with a maximum of two bit transitions. In images related to eye diseases, significant textural changes mostly occur at the local scale, and the use of small-radius LBP provides sufficient representation. While larger radius values or multi-scale LBP approaches can provide broader contextual information, they have disadvantages such as increased feature size and computational cost. Since the aim of this study was to create a lightweight texture representation complementing RGB-based deep features, the standard Uniform LBP (P = 8, R = 1) was used. In addition, other texture descriptors such as Gabor filters or Gray-Level Co-occurrence Matrix (GLCM) are preferred in the literature. However, since these methods have disadvantages such as higher computational costs or requiring more complex parameter settings, LBP was used in this study for its computational efficiency.

### 4.5. Feature Fusion and Classification

A new feature map is created by combining the feature vectors obtained from the RGB and LBP branches. The RGB branch represents visual features (color, shape, edge patterns) with a 1792-dimensional feature map obtained from the EfficientNetV2-S model, while the LBP branch represents texture features (surface patterns, local density differences) with a 1024-dimensional feature map.

Classification is performed with the forward method of the MidFusionEfficientV2. This stage allows the model to make a decision by combining what it has learned from both RGB and LBP branch. For example, knowing the blurriness of cataract in an image is looked at by the RGB branch while irregularities on superficial tissue is scrutinized through LBP to make correct classification.

### 4.6. Training

**Figure 4:**
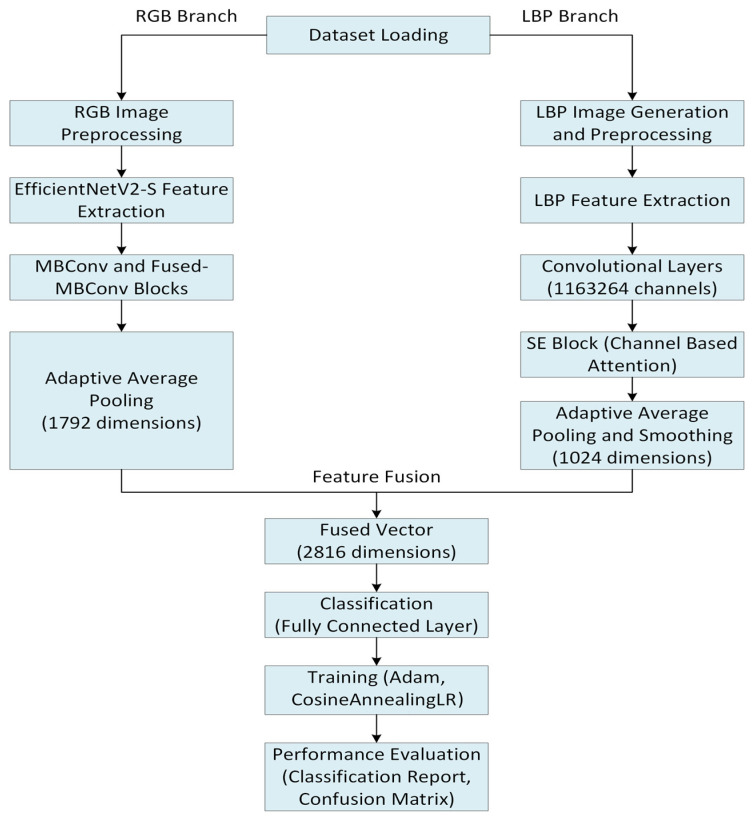
Detailed flow chart of the proposed method.

The proposed model is trained for 25 epochs. The model is trained with the Adam optimizer. A learning rate of 0.0002 is used for EfficientNetV2-S and 0.002 for the LBP branch and the fully connected layer. This setting allows precise updating of the pre-trained weights and faster learning of new layers. During training, the Cosine Annealing Learning Rate Scheduler method provides a more balanced learning. The flowchart of the proposed model is shown in Figure 4. The flow chart of the proposed model is given in Figure 4.

The fusion we propose is performed at the feature level. Feature maps obtained from the EfficientNetV2-S-based RGB branch were converted into a 1792-dimensional feature vector using global average pooling. The LBP-based convolution branch, after adaptive average pooling, produces a 1024-dimensional feature representation. These two feature representations are combined using the concatenation operator, and the resulting combined feature vector is then transferred to the fully coupled classification layer. The fusion process follows the feature extraction phase. However, since this process is performed before the classification layer, this approach is referred to in the literature as feature-level (mid-level) fusion.

## 5. Results

Artificial intelligence techniques have been used in various fields in recent years, particularly in the biomedical sector. In this study, artificial intelligence techniques were used for the detection of eye diseases. Our model training process involved conducting all experiments under the same experimental framework. The batch size was set to 8 during training. The Cross-Entropy loss function was used for model training. The learning rate was set to 0.0002 for the EfficientNetV2-S backbone and 0.002 for the LBP branch and fully connected layer. To ensure a balanced decrease in the learning rate during training, the Cosine Annealing Learning Rate Scheduler was used, with the scheduler parameter set to T_max = 25. The training process was completed in 25 epochs. To ensure reproducibility in the experimental process, the random seed value was fixed. Additionally, the dataset was stratified into training (70%), validation (15%), and testing (15%) subsets. Furthermore, the same training protocol and data-splitting strategy were applied to ensure a fair comparison of all baseline models against our proposed model. In the experimental study, the performance of the proposed MidFusionEfficientV2 model was evaluated on a dataset of five classes of eye diseases from the Mendeley Data platform. In addition, the performance of the proposed model is compared with 6 different deep learning models, namely ResNetV2, ConvNeXt, DenseNet-121, EfficientNet-B1, MobileNetV3 Large, and EfficientNetV2-S. During training, the number of epochs for all models was 25, and the Cosine Annealing Learning Rate Scheduler method was used. The class-wise performances of ResNetV2, ConvNeXt, DenseNet-121, EfficientNet-B1, MobileNetV3 Large, EfficientNetV2-S, and the proposed MidFusionEfficientV2 according to precision, recall, and F1-score metrics are given in Table 1, Table 2, Table 3, Table 4, Table 5, Table 6, and Table 7, respectively.

Table 1 shows that Class-Based Metrics for ResNetV2 are particularly successful in the normal class. The class in which the ResNetV2 model is least successful is Uveitis, with a Precision of 0.88. The accuracy value of the ResNetV2 model is 0.95. Class-based performance metrics for the ConvNeXt model are presented in Table 2.

Table 2 shows that the ConvNeXt model performed best in the Normal class, achieving a precision of 1.00. The class in which the ConvNeXt model achieved the lowest performance was Uveitis, with a precision of 0.80. The overall accuracy rate of the ConvNeXt model was 0.93. The class-based performance metrics of the DenseNet-121 model, another model used in the study, are shown in Table 3.

When examining the class-based performance metrics of the DenseNet-121 model, it is observed that the model performed best on the Normal class and worst on the Uveitis class. Following the Normal class, the model performed most successfully in the Cataract class. The accuracy of the DenseNet-121 model on the relevant dataset is 0.92. The class-based performance measurement metrics of EfficientNet-B1, another model used in this study, are presented in Table 4.

Examining the class-based performance metrics of the EfficientNet-B1 model presented in Table 4, it is evident that the model is most successful on the Normal class, with a precision of 0.98. This is followed by Conjunctivitis, Cataract, Uveitis, and Eyelid, respectively. The accuracy value of the EfficientNet-B1 model is 0.90. The class-based performance metrics of the MobileNetV3 Large model are presented in Table 5.

Table 5 shows that the MobileNetV3 Large model performed best in the Eyelid class with a precision of 0.99. However, the MobileNetV3 Large model performed poorly on the Uveitis class, which was the most confusing for the model. The accuracy achieved by the MobileNetV3 Large model was 0.94. The final model used in the study for comparison of the proposed model’s performance is EfficientNetV2-S. The class-based performance evaluation metrics of the EfficientNetV2-S model are presented in Table 6.

Table 6 shows that the EfficientNetV2-S model performed best in the normal class. This was followed by cataract, eyelid, uveitis, and conjunctivitis, respectively. The accuracy value of the EfficientNetV2-S model was 0.94. The performance metrics of the Proposed MidFusionEfficientV2 model are presented in Table 7.

When the tables were examined, it was observed that the proposed MidFusionEfficientV2 model exhibited a clear superiority compared to all the comparative architectures in class-based performance analyses. The model achieved a remarkably high success rate of 98% in terms of overall accuracy, which was higher than all other models. Considering that strong reference models like ResNetV2 and EfficientNetV2-S achieved accuracy values of 95% and 94% respectively, the results provided by MidFusionEfficientV2 are noteworthy. The proposed model’s ability to differentiate between classes is evenly distributed, particularly standing out with a high F1 score (94%) in the Uveitis class, which has a high level of difficulty. For this class, most models perform below 80% or barely at the threshold, while the proposed model achieved 91% precision and 97% recall, demonstrating effective learning even in classes with few examples. Table 8 presents the ablation results.

**Figure 5:**
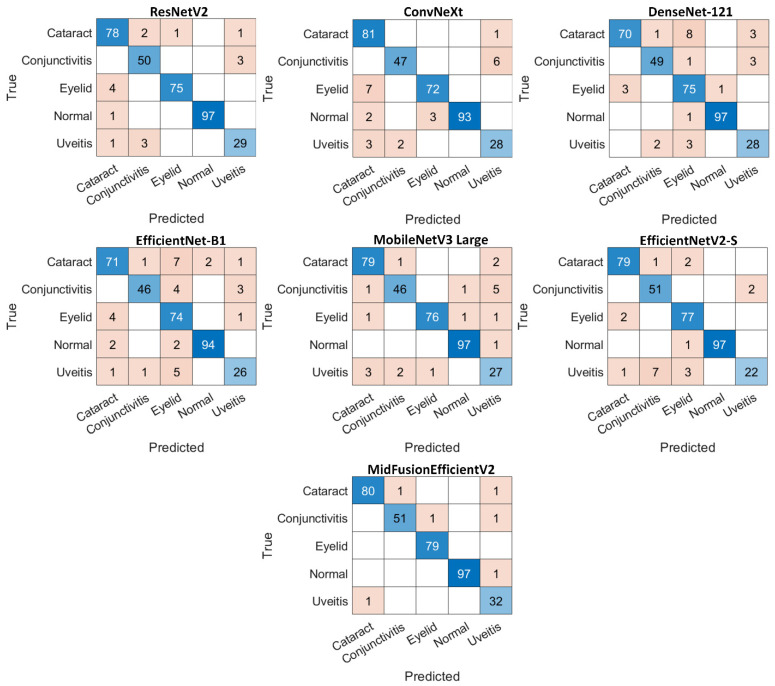
Confusion matrices for all models.

To better understand the images that the models correctly predict based on class, the confusion matrices of all models are given in Figure 5.

Looking at the confusion matrix of the proposed MidFusionEfficientV2, it is seen that the predictions for each class are quite accurate. In the Cataract class, it correctly classified 80 out of 82 samples, with only two errors. In the Conjunctivitis class, it delivered a very balanced performance with 51 correct predictions and only two errors. In the Eyelid class, it correctly predicted all 79 samples. In the Normal class, it achieved 99% accuracy with only one error. For the most challenging class, Uveitis, it correctly predicted 32 out of 33 samples, which is the highest number of correct classifications compared to all other models.

When analyzing other models, the ResNetV2 model showed 3 mispredictions in the Uveitis class and significant error rates in some classes (e.g., Eyelid). ConvNeXt showed 6 mispredictions in the Conjunctivitis class, demonstrating the model’s inability to distinguish between these classes. The DenseNet-121 model showed a significant deficiency in this area, misclassifying 12 instances in the Cataract category. EfficientNet-B1 exhibited a significant number of errors in the cataract, eyelid, conjunctivitis, and uveitis classes, suggesting that the model struggles to distinguish more complex textural variations. The MobileNetV3 Large model also exhibited suboptimal performance, exhibiting six errors, particularly in the Uveitis class. In the EfficientNetV2-S model, the Uveitis class was the most misclassified class, recording a total of 11 errors.

**Figure 6:**
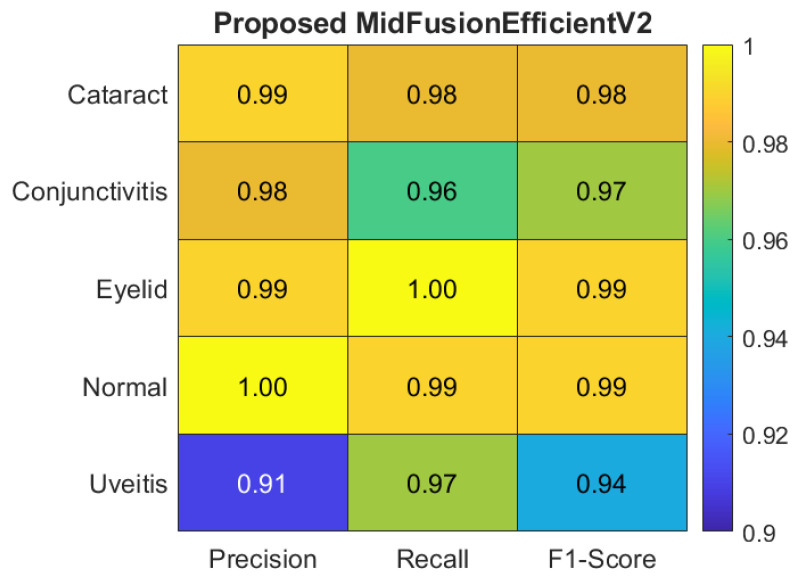
Heatmap of class-based metrics of the proposed MidFusionEfficientV2.

**Figure 7:**
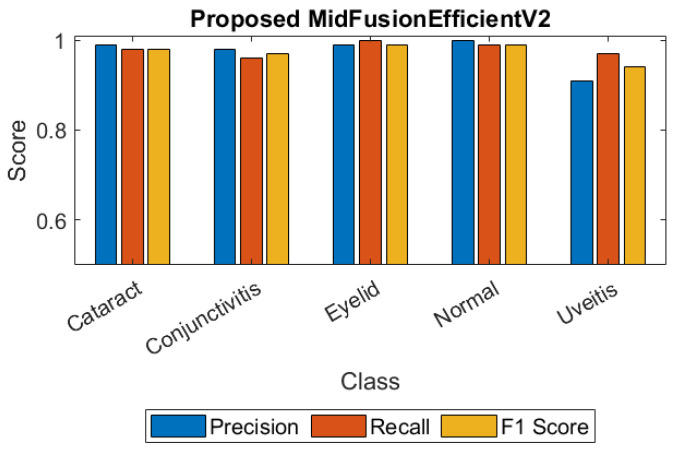
Bar chart of class-based metrics of the proposed MidFusionEfficientV2.

For a clearer understanding of the class-based metrics of the proposed model, the heatmap and bar chart are given in Figure 6 and Figure 7 respectively.

**Figure 8:**
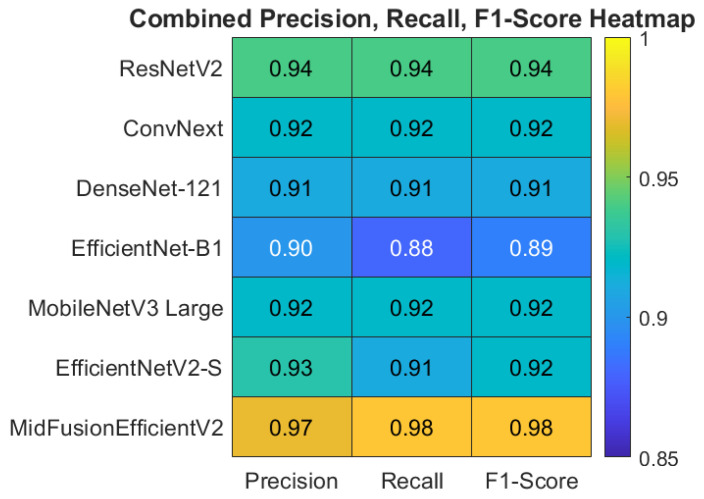
Heatmap of Precision, Recall And F1-Score Based on Macro Avg For All Models.

**Figure 9:**
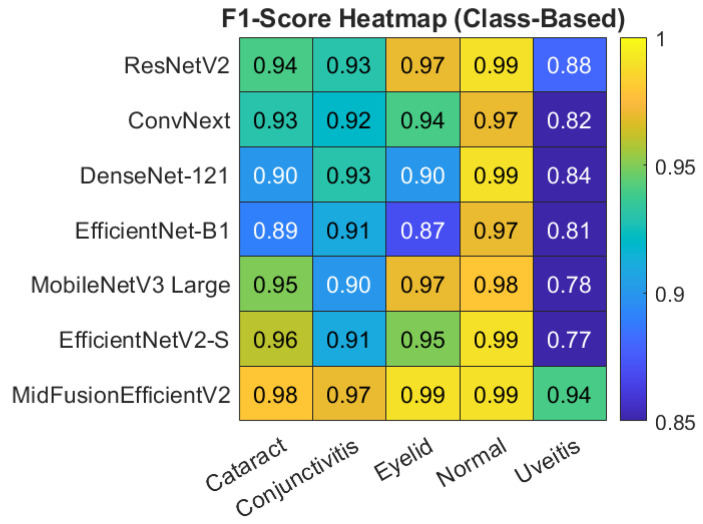
Heatmap of class-based F1-score for all models.

**Figure 10:**
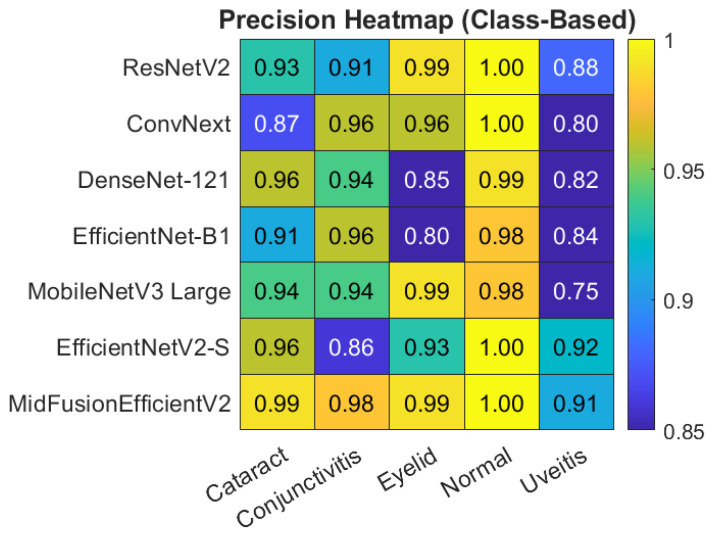
Heatmap of class-based Precision for all models.

**Figure 11:**
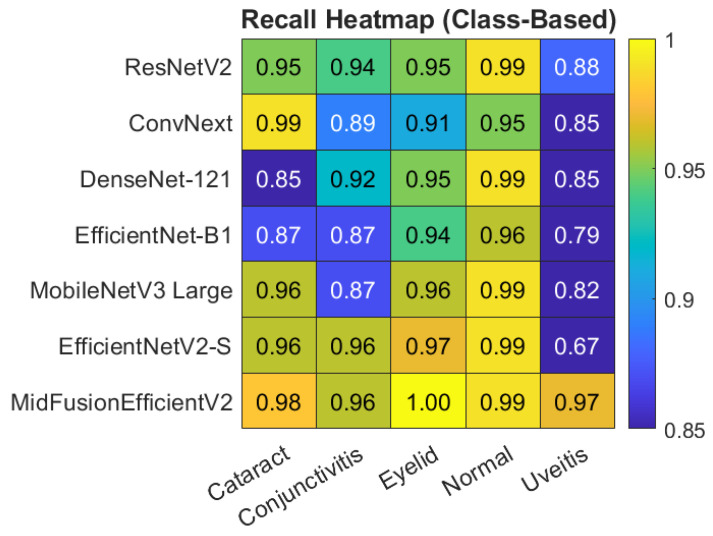
Heatmap of class-based Recall for all models.

Figure 8 shows the heatmap of Precision, Recall and F1-Score on macro avg basis obtained by all models. Also, the class-based heatmaps of F1-Score, Precision and Recall obtained by all models are given in Figure 9, Figure 10 and Figure 11, respectively.

Summaries of publications in the literature similar to this study are presented in Table 9. Examining the table, the MidFusionEfficientV2 model proposed in this study achieved the highest accuracy rate (98%) among current studies in the literature. In particular, the mid-level fusion of the EfficientNetV2-S-based RGB branch and the LBP+SE-based texture branch, combined with the evaluation of both color and texture information, significantly improved classification performance. An examination of the table reveals that the success rates of the methods used in studies published in recent years, in particular, range from 95.2% to 98%. However, many of these studies were evaluated on datasets with only 2, 3, or 4 classes. In contrast, the proposed method achieved a remarkably high accuracy rate of 98% despite being tested on a more complex dataset consisting of five classes. This demonstrates both the classification power and generalizability of the proposed method.

Table 9 shows that the proposed model achieved competitive results.

**Figure 12:**
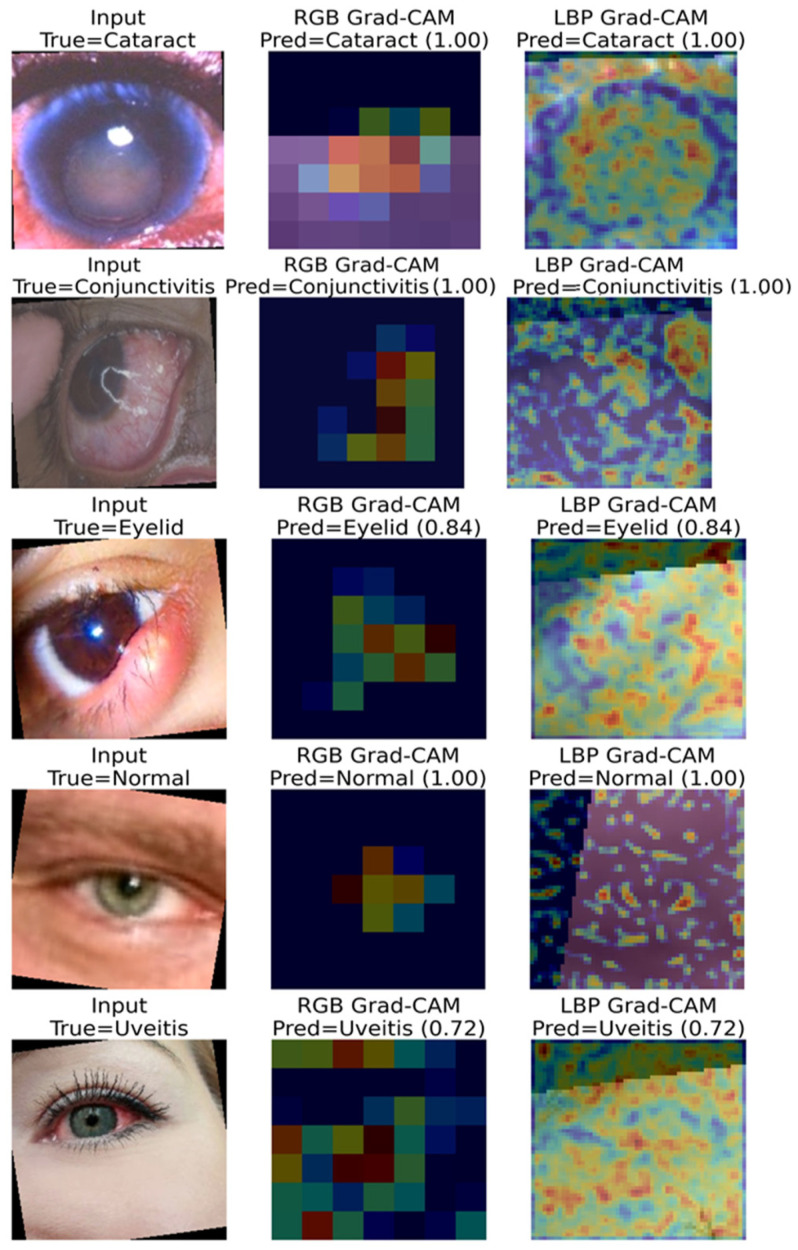
Grad-CAM visualizations of the RGB and LBP branches for representative test samples.

Figure 12 shows the Grad-CAM-based explainability results of the proposed RGB–LBP mid-level fusion model.

Examining Figure 12, each row shows the input image for the relevant class, along with activation maps obtained from the RGB and LBP arms. It is clearly seen that in cataract, conjunctivitis, and uveitis samples, the RGB arm focuses on disease-specific macroscopic regions, while the LBP arm captures more widespread tissue patterns. The absence of a distinct focus in the normal class indicates that the model correctly learned when pathological findings were absent. These results demonstrate that RGB focuses on morphological features and LBP on textural features, showing significant visual explainability regarding the combined interpretability of the two representations.

## 6. Conclusions

This study proposes MidFusionEfficientV2, a hybrid deep learning model for automated classification of eye diseases. Unlike traditional RGB-based approaches, this model utilizes a dual-branch architecture that simultaneously evaluates color and texture features. By combining textural features obtained through LBP transformation with the global visual features of EfficientNetV2-S, the model’s ability to distinguish complex situations is enhanced. In particular, SE blocks integrated into the LBP branch improve the model’s performance by making texture features more meaningful and distinctive. The dataset used to train the model consists of eye disease images from five classes obtained from the Mendeley data platform. Experimental studies have shown that the proposed model outperforms frequently used models in the literature, including ResNetV2, ConvNeXt, DenseNet-121, EfficientNet-B1, MobileNetV3 Large, and EfficientNetV2-S. The model achieved a 98% overall accuracy, 97% recall rate, and 94% F1 score. These results demonstrate that the model performs well even with small sample sizes and challenging classes. However, our study also has some limitations. Our study was tested on a public dataset. Another limitation is that the reported accuracy values are based on a single experimental study. Our future work will include repeated experiments with different random seeds and resampling-based uncertainty estimates to provide a more reliable assessment of the proposed model’s stability. Furthermore, we will further strengthen the applicability of the proposed model to real-world scenarios by testing it with different datasets and additional eye disease classes.

## Figures and Tables

**Table 1:** Class-Based Metrics for ResNetV2.

Class	Precision	Recall	F1-Score	Support
Cataract	0.93	0.95	0.94	82
Conjunctivitis	0.91	0.94	0.93	53
Eyelid	0.99	0.95	0.97	79
Normal	1.00	0.99	0.99	98
Uveitis	0.88	0.88	0.88	33
Macro avg.	0.94	0.94	0.94	345
Weighted avg.	0.95	0.95	0.95	345
Accuracy	0.95			345

**Table 2:** Class-Based Metrics for ConvNeXt.

Class	Precision	Recall	F1-Score	Support
Cataract	0.87	0.99	0.93	82
Conjunctivitis	0.96	0.89	0.92	53
Eyelid	0.96	0.91	0.94	79
Normal	1.00	0.95	0.97	98
Uveitis	0.80	0.85	0.82	33
Macro avg.	0.92	0.92	0.92	345
Weighted avg.	0.93	0.93	0.93	345
Accuracy	0.93			345

**Table 3:** Class-Based Metrics for DenseNet-121.

Class	Precision	Recall	F1-Score	Support
Cataract	0.96	0.85	0.90	82
Conjunctivitis	0.94	0.92	0.93	53
Eyelid	0.85	0.95	0.90	79
Normal	0.99	0.99	0.99	98
Uveitis	0.82	0.85	0.84	33
Macro avg.	0.91	0.91	0.91	345
Weighted avg.	0.93	0.92	0.92	345
Accuracy	0.92			345

**Table 4:** Class-Based Metrics for EfficientNet-B1.

Class	Precision	Recall	F1-Score	Support
Cataract	0.91	0.87	0.89	82
Conjunctivitis	0.96	0.87	0.91	53
Eyelid	0.80	0.94	0.87	79
Normal	0.98	0.96	0.97	98
Uveitis	0.84	0.79	0.81	33
Macro avg.	0.90	0.88	0.89	345
Weighted avg.	0.91	0.90	0.90	345
Accuracy	0.90			345

**Table 5:** Class-Based Metrics for MobileNetV3 Large.

Class	Precision	Recall	F1-Score	Support
Cataract	0.94	0.96	0.95	82
Conjunctivitis	0.94	0.87	0.90	53
Eyelid	0.99	0.96	0.97	79
Normal	0.98	0.99	0.98	98
Uveitis	0.75	0.82	0.78	33
Macro avg.	0.92	0.92	0.92	345
Weighted avg.	0.94	0.94	0.94	345
Accuracy	0.94			345

**Table 6:** Class-Based Metrics for EfficientNetV2-S.

Class	Precision	Recall	F1-Score	Support
Cataract	0.96	0.96	0.96	82
Conjunctivitis	0.86	0.96	0.91	53
Eyelid	0.93	0.97	0.95	79
Normal	1.00	0.99	0.99	98
Uveitis	0.92	0.67	0.77	33
Macro avg.	0.93	0.91	0.92	345
Weighted avg.	0.95	0.94	0.94	345
Accuracy	0.94			345

**Table 7:** Class-Based Metrics for Proposed MidFusionEfficientV2.

Class	Precision	Recall	F1-Score	Support
Cataract	0.99	0.98	0.98	82
Conjunctivitis	0.98	0.96	0.97	53
Eyelid	0.99	1.00	0.99	79
Normal	1.00	0.99	0.99	98
Uveitis	0.91	0.97	0.94	33
Macro avg.	0.97	0.98	0.98	345
Weighted avg.	0.98	0.98	0.98	345
Accuracy	0.98			345

**Table 8:** Ablation study evaluating the individual contributions of the LBP branch, SE module, and mid-level fusion strategy.

Model	Accuracy	Macro-F1	Weighted-F1
RGB-only (EfficientNetV2-S)	0.94	0.92	0.94
LBP-only	0.80	0.75	0.79
RGB+LBP (No SE)	0.95	0.93	0.95
RGB+LBP+SE (Proposed)	0.98	0.98	0.98

**Table 9:** Comparative results of eye disease classification studies.

Authors	Year	Model Used	Target Diseases	Accuracy (%)	Dataset
Mondal et al. [50]	2022	VGG-19, ResNet-50, Inception-v3	Conjunctivitis (Viral, Bacterial) vs Normal	95.2	210 eye images (2 classes)
Zannah et al. [51]	2024	EfficientNet + PCA + SVM	Cataract, Diabetic Retinopathy, Glaucoma, and Normal	95.33	5181 fundus images (4 classes)
Sattigeri et al. [52]	2022	Custom CNN	Uveitis, Conjunctivitis, Cataract, Bulging Eyes, Crossed Eyes	96	1200 eye images (5 classes)
Bitto & Mahmud [53]	2022	VGG-16, ResNet-50, Inception-v3	Normal, Conjunctivitis, Cataract	97.08	2250 eye images (3 classes)
Nguyen & Lin [33]	2024	Hybrid CNN (segment-based analysis)	Cataract, Normal	97.12	1388 fundus images (2 classes)
Khalid et al. [35]	2024	MobileNet + EfficientNet (CAD-EYE)	Diabetic Retinopathy, Hypertensive Retinopathy, Glaucoma, and Contrast-related	98	65,871 fundus images (4 classes)
Our Proposed Model	-	EfficientNetV2-S + LBP + SE (Hybrid CNN)	Uveitis, Conjunctivitis, Cataract, Eyelid Drooping, Normal	98	2298 eye images (5 classes)

## Data Availability

The data presented in this study are openly available [Mendeley] [https://doi.org/10.17632/N9ZP473WFW.1] [36].
